# Clinical characteristics of fetal and neonatal outcomes in twin pregnancy with preeclampsia in a retrospective case–control study: A STROBE-compliant article

**DOI:** 10.1097/MD.0000000000005199

**Published:** 2016-10-28

**Authors:** Ting Yuan, Wei Wang, Xue-Lan Li, Chun-Fang Li, Chao Li, Wen-Li Gou, Zhen Han

**Affiliations:** a Department of Obstetrics & Gynecology, First Affiliated Hospital of Xi’an Jiaotong University College of Medicine; b Department of Public Health, Xi’an Jiaotong University College of Medicine, Shaanxi, China.

**Keywords:** birth weight, outcome, preeclampsia (PE), twin pregnancy

## Abstract

The aim of our study was to compare the clinical characteristics of fetal and neonatal outcomes in twin pregnancies between women with preeclampsia (PE) and those with normotension in a Chinese population.

There were 143 preeclamptic women and 367 normotensive women with twin pregnancies included in this retrospective case–control study. The baseline characteristics and perinatal outcomes were collected and compared between the groups. Multiple logistic regression and linear regression were used to assess the correlations between PE and the outcomes.

Significant increases were observed in the frequencies of preterm delivery (OR = 2.75, *P* < 0.001), iatrogenic preterm birth (OR = 3.52, *P* < 0.001), and IUGR (OR = 2.94, *P* = 0.001) in the PE group, and the PE group had more than a 2-fold risk of adverse neonatal outcomes. Preeclamptic twin neonates had lower birth weights (β = −147.34, *P* = 0.005; β = −169.47, *P* = 0.001). The comparison on the discordance of intertwin weight was not significantly different.

Twin pregnancies with PE are associated with worse perinatal outcomes. The adverse outcomes of preeclamptic twin pregnancies may be associated with lower birth weights rather than the discordance of the intertwin weight, which requires further confirmation. The results may provide helpful references for better clinical assessments, evaluations of prognosis, and a deeper understanding of preeclamptic twin pregnancies.

## 1 Introduction

Preeclampsia (PE) is a common and severe form of hypertensive disorder complicating pregnancy (HDCP) and remains a leading direct cause of maternal and neonatal mortality and morbidity.^[1]^ The prevalence of PE was approximately 4.0% and 2.88% of all pregnancies worldwide and in mainland China, respectively.^[^2 3^]^

Recently, multiple studies have focused on the incidence and risk factors of twin pregnancies with PE,^[^4–8^]^ but inadequate data exist regarding the clinical characteristics and perinatal outcomes of twin pregnancies with PE.^[^9–12^]^ It is acknowledged that PE increases the risk of adverse maternal–neonatal outcomes mainly from data of singleton pregnancies.^[^1 2 13 14^]^ However, twin pregnancies with hypertensive disorders were unexpectedly shown to have better neonatal outcomes than normotensive women in twin pregnancy.^[9]^ Therefore, subjective judgments or deductions could not be made to determine the perinatal outcomes in preeclamptic twin pregnancies, and robust statistical evidence needs to be provided. Meanwhile, a small amount of data on this point has been retrieved in a Chinese population.

The aim of our study was to compare the clinical characteristics of fetal–neonatal outcomes in twin pregnancies between women with PE and those with normotension, and the conclusions of this study may be helpful for better clinical assessments and evaluations of prognosis.

## 2 Materials and methods

### 2.1 Subjects

This was a retrospective case–control study that was conducted at the First Affiliated Hospital of Xi’an Jiaotong University, Shaanxi, China, from January 2008 to March 2015. Women who had a twin pregnancy with a clear confirmation of chorionicity were included; those who had PE were included in the case group, and those who were normotensive were included in the control group.

The diagnosis of PE included the following: severe PE (diastolic blood pressure ≥ 110 mm Hg, systolic blood pressure ≥ 160 mm Hg, proteinuria from none to positive, elevated serum creatinine and transaminase levels, obvious fetal growth restriction (FGR), multiorgan disturbances, such as headache, visual disturbance, upper abdominal pain, oliguria, convulsion, thrombocytopenia, and pulmonary edema) and nonsevere PE (diastolic blood pressure <110 mm Hg, systolic blood pressure <160 mm Hg, proteinuria from none to positive, no multiorgan disturbances or only minimal serum transaminase elevation). Nonsevere PE includes “mild” and “moderate” hypertension, which is not specifically defined. Overt proteinuria may not be a necessary element to characterize the PE syndrome.^[15]^ Hypertension was defined as a systolic blood pressure ≥ 140 mm Hg and/or a diastolic blood pressure ≥90 mm Hg on at least 2 occasions at a 6-hour interval.

We excluded women with any of following items in the current pregnancy: gestational hypertension, chronic hypertension, or eclampsia; acute internal or surgical diseases; triplet or quadruplet pregnancy; and chorionicity that was unclear. Only cases with a cotwin live birth were included in the analyses for the neonatal outcomes.

The study was approved by the ethics and research committee of the First Affiliated Hospital of Xi’an Jiaotong University (No. XJTU1AF2015LSL-073), and it conformed to the provisions of the Declaration of Helsinki (as revised in Brazil 2013). A written informed consent was not necessary for this retrospective study, and patient anonymity was preserved.

### 2.2 Clinical evaluation

The gestational age was calculated using the last menstruation period (LMP) or confirmed by the first-trimester ultrasound. The baseline characteristics and maternal–neonatal outcomes were obtained from the medical records. The determination of twin chorionicity was clinically based on an ultrasound evaluation, including the gestational sac number at early pregnancy, the placental number, the fetal sex, “T” sign or “λ” sign,^[16]^ and it was confirmed by a placenta examination after delivery. Placental anomalies included placental abruption, previa, increta, and adherent. Umbilical cord anomalies included prolapsus, torsion, and knots. Abnormal amniotic fluid referred to at least 1 fetus with polyhydramnios (maximum vertical pocket more than 8 cm) or oligoamnios (maximum vertical pocket was less than 2 cm).^[17]^ The umbilical artery Doppler flow spectrum was recorded from a free floating portion of the umbilical cord during minimal fetal activity and the absence of fetal breathing, and the average pulsatility index (PI) value of the cotwin was used for the final analysis. The values of the amniotic fluid and PI for the analysis were from the last ultrasound before delivery. Intrauterine growth restriction (IUGR) was defined as at least 1 fetus with a birth weight less than the 10th percentile for the gestational age, and the birth weight percentiles for gestational age were determined by the Ananth twin reference curve.^[18]^ Single intrauterine fetal death (sIUFD) referred to the death of 1 twin in the uterine. Discordance of the intertwin birth weight or the estimated fetal weight (EFW) was defined as [(larger baby weight – smaller baby weight)/larger baby weight] (%).^[19]^ The diagnoses of small for gestational age (SGA), low birth weight (LBW), very low birth weight (VLBW), and extremely low birth weight (ELBW) were each defined according to the William obstetrics.^[15]^

### 2.3 Statistical analyses

The data collected from the study subjects were verified and double entered into a data management system. The parameters are presented as the mean ± SD or the median (range) and percentages, as appropriate. The statistical analyses were performed using the Chi-square, Fisher exact, Student *t*, Mann–Whitney *U* tests, as appropriate. Multiple logistic regression and linear regression were used to determine the odds ratio (OR) or β and 95% confidence interval (CI) of some of the selected outcomes among the women with or without PE, and the potential confounding factors were included in the analysis. All of the reported *P* values were 2-tailed, and values <0.05 were considered statistically significant. The statistical analyses were performed using SPSS version 23.0 (IBM, Armonk, NY).

## 3 Results

A total of 585 twin pregnancies were retrieved in the hospital information system, and 75 cases were excluded because they did not meet the inclusion criteria or because there were incomplete maternal or neonatal records. As a result, 143 twin pregnancies with PE and 367 twin pregnancies with normotension were included in the final analysis.

The baseline characteristics and maternal–fetal outcomes of the PE and normotension groups in the twin pregnancies are described in Table 1. There were no significances in the baseline characteristics. The total number of preterm deliveries was greater in the twin pregnancies with PE (*P* = 0.003) and the iatrogenic type, and those deliveries before 33^+6^ weeks were even more common in this group (*P* = 0.034 and *P* < 0.001, respectively), and more IUGR occurred in the preeclamptic women (*P* < 0.001). They were also more inclined to be hospitalized at an earlier gestational age and deliver earlier (*P* = 0.010 and *P* = 0.005, respectively), and the total hospitalized duration of the preeclamptic women was also longer (*P* < 0.001) (Table 1).

**Table 1:**
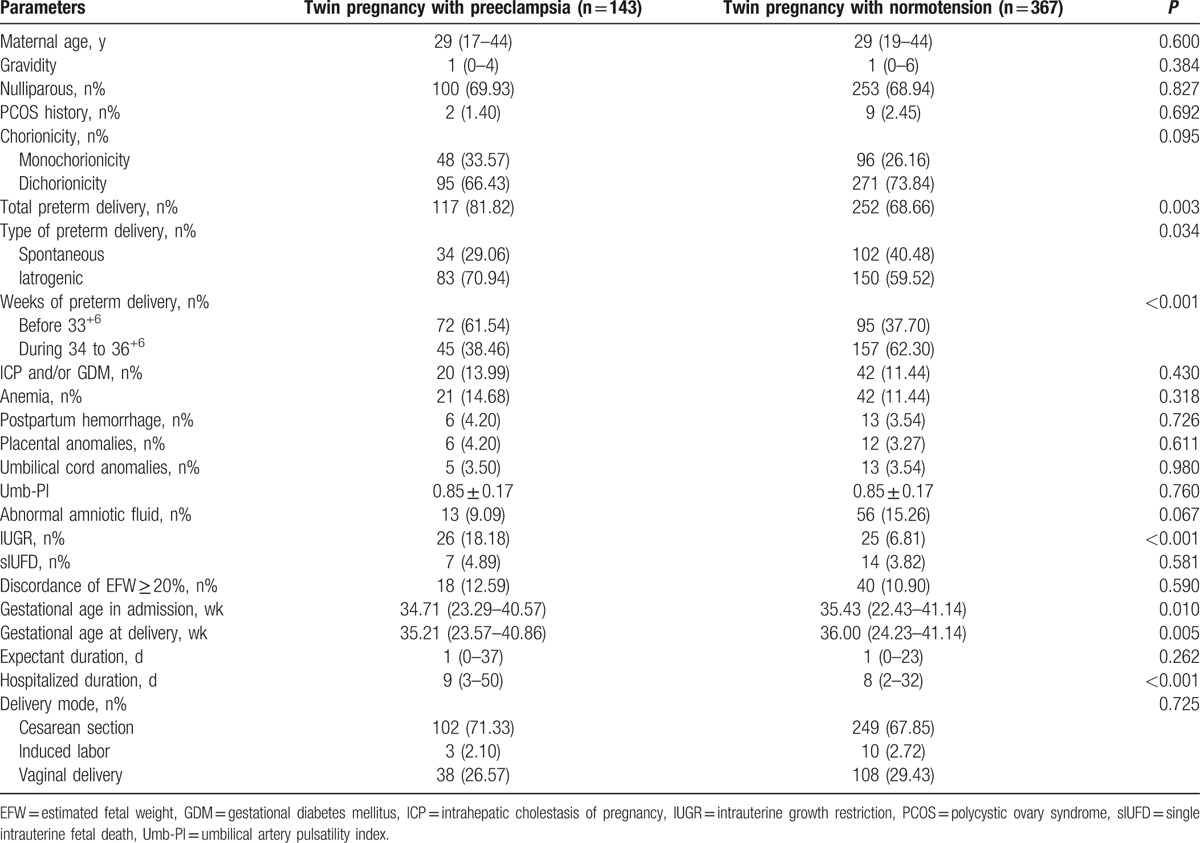
The baseline characteristics and maternal–fetal outcomes of preeclampsia and normotension group in twin pregnancy.

Next, the neonatal outcomes of the cotwin live births between the 2 groups were subsequently compared in Table 2. Cotwin live birth in the PE and normotension groups were 134 (268 neonates, 93.71%) and 341 (682 neonates, 92.91%), respectively; however, there were no significant differences between the 2 groups (*P* = 0.751). The larger baby weight, smaller baby weight, and percentile of smaller baby weight were significantly lower in the PE group than the normotension group (*P* = 0.007, *P* = 0.001, and *P* = 0.006, respectively). However, the comparison on the discordance of the intertwin weight was not significant. A series of neonatal complications were more likely to occur in the PE group, such as intracranial hemorrhage (*P* = 0.043), neurological complications (*P* < 0.001), ischemic myocardial injury (*P* < 0.001), and acid-base and electrolyte disturbance (*P* = 0.020). Moreover, the number of neonates diagnosed at SGA, LBW, and with an Apgar score 1 minute < 7, were even more in the PE group (*P* < 0.001, *P* = 0.002, and *P* = 0.037, respectively). In addition, more neonates were transferred to the neonatal intensive care unit (NICU) and were hospitalized for longer durations in the PE group (*P* < 0.001 for both) (Table 2).

**Table 2:**
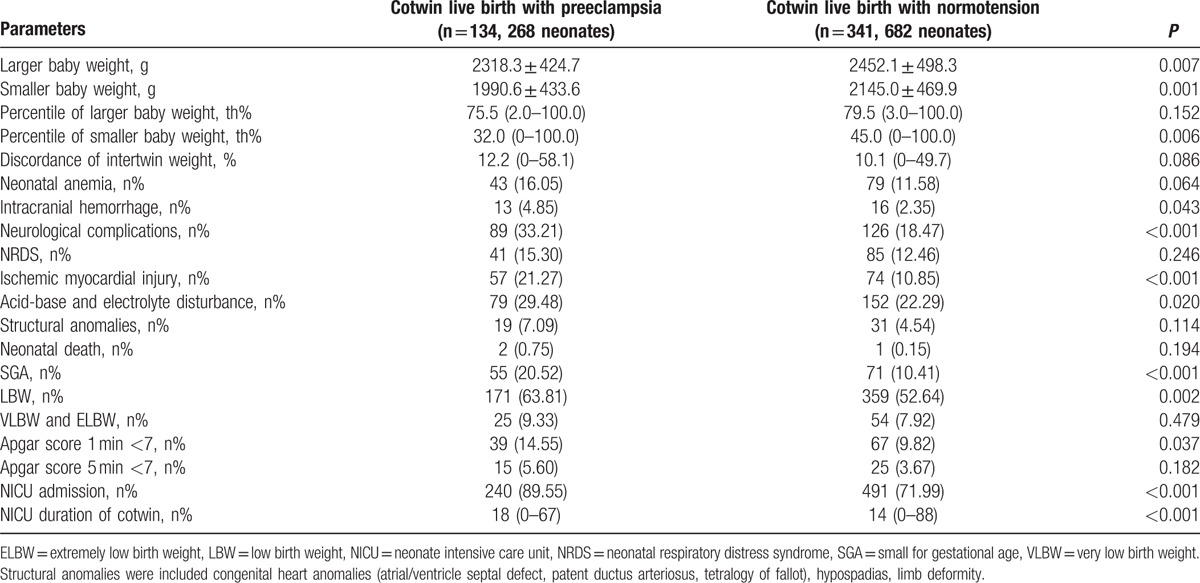
The neonatal outcomes of cotwin live birth in preeclampsia and normotension group.

According to the multiple logistic regression adjusted for potential covariates, some of the selected maternal–fetal and neonatal outcomes remained statistically significant. There were significant increases in the frequencies of preterm birth (OR = 2.75, 95% CI = 1.69–4.47, *P* < 0.001), iatrogenic preterm birth (OR = 3.52, 95% CI = 2.73–5.53, *P* < 0.001), and IUGR (OR = 2.94, 95% CI = 1.56–5.53, *P* = 0.001) in twin pregnancies with PE. Moreover, the preeclamptic neonates were had more than a 2-fold risk of adverse neonatal outcomes (Table 3).

**Table 3:**
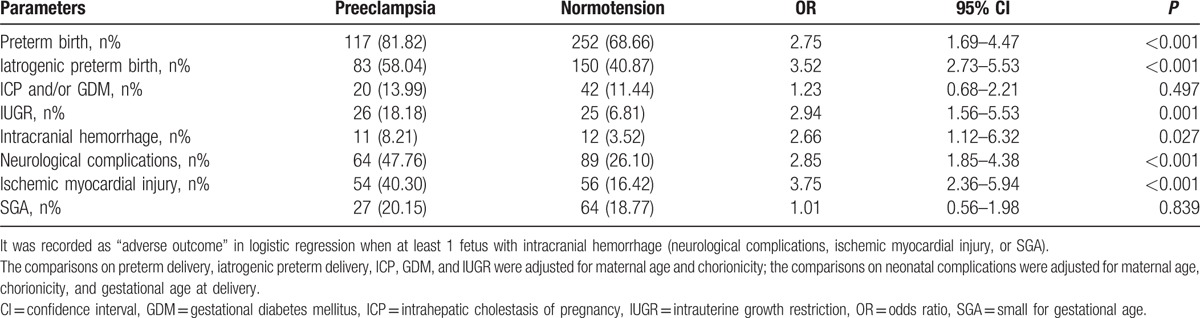
Selected perinatal outcomes in twin pregnancy with preeclampsia compared with normotension group adjusted for potential covariates.

According to the multiple linear regression adjusted for potential covariates, the larger and smaller baby weights were still significantly lower in the preeclamptic group (β = −147.34, 95% CI = −249.33 to −45.35, *P* = 0.005; β = −169.47, 95% CI = −267.98 to −70.97, *P* = 0.001). No significances were observed in the comparisons of the gestational age at delivery and the discordance of the intertwin weight (Table 4).

**Table 4:**
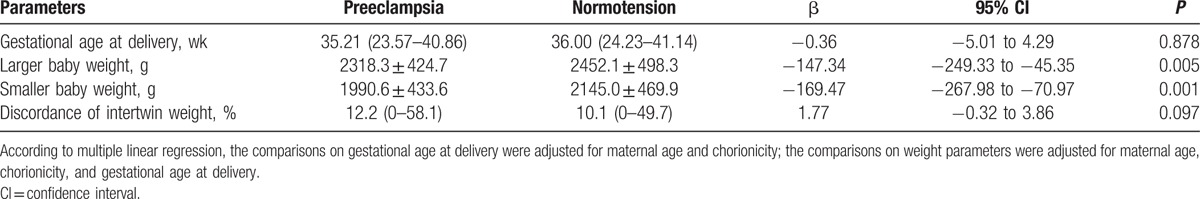
Neonatal weight outcomes in cotwin live birth with preeclampsia compared with normotension group adjusted for potential covariates.

## 4 Discussion

### 4.1 Worse perinatal outcomes in preeclamptic twin pregnancies

Compared with normotensive women with twin pregnancies, there were significant correlations between PE and adverse perinatal outcomes. PE is currently acknowledged as a chronic placental ischemic disease, resulting from inadequate modified spiral arteries. Placental dysfunction and hypoperfusion play an important pathophysiological role and are considered to be associated with adverse perinatal outcomes, including reduced birth size, IUGR, preterm birth, prenatal loss, and a broad range of complications.^[^1 20–22^]^ Based on our results, we speculate that the pathophysiological process of PE in twin pregnancies may be similar to that in singleton pregnancies.

However, the relationship between PE and IUGR in twin pregnancies is a point that deserves further discussion. Fox et al^[23]^ proposed that there was no correlation between PE and IUGR in twin pregnancies from a cohort of 578 patients. Women with PE were not more likely to have growth restricted neonates, and women with growth restricted neonates were not more likely to have PE. There was a strong link between PE and reduced fetal growth in singleton pregnancies, according to multiple previous studies as we mentioned above. However, no correlation between these 2 entities in twin pregnancies might reveal different pathological mechanisms of PE and IUGR in this distinctive condition. It is possible that the low birth weight (LBW) in the twin pregnancies is mainly due to the inability of the environment to meet the nutritional needs in multiple pregnancies rather than an ischemic placenta in PE. Meanwhile, risk factors associated with PE or IUGR in singleton pregnancy had inability to predict PE or IUGR in twin pregnancy (aside from obesity and egg donation for PE).^[^4 23 24^]^

Interestingly, a retrospective cohort study from Chinese twin pregnancies revealed that PE occurred more frequently in selective IUGR (sIUGR) pregnancies than in normal growth twin pregnancies, and sIUGR was associated with increased odds of developing severe PE in twin pregnancies.^[25]^ These 2 studies and ours were coming from different perspectives to look into the correlations between PE and IUGR in twin pregnancies. The different definitions of IUGR and the diverse twin growth curve used in the studies may account for these different results. The real pathological correlations between PE and IUGR both in twin and singleton pregnancies should be confirmed by further research.

According to previous studies, PE is one of the risk factors for preterm birth.^[^26 27^]^ The present study also demonstrated that the occurrence of PE in twin pregnancies was an important risk factor for preterm birth and the iatrogenic type after adjusting for potential covariates. Preeclamptic women were more likely to have a preterm delivery due to medical complications, and the majority of the women delivered at an early gestational week. PE was also found to be one of the most frequent indications for labor induction of iatrogenic late preterm births in the other studies.^[28]^ It was additionally suggested that short-term neonatal outcomes differed by the etiology (spontaneous or iatrogenic) of preterm birth,^[29]^ iatrogenic late preterm birth increased the risk of NICU admission and respiratory disorders in neonates in comparison to the spontaneous type.^[28]^ Although we have observed more iatrogenic preterm births and worse adverse neonatal outcomes in preeclamptic mothers, the correlation between them has not been demonstrated yet. If the iatrogenic preterm birth is confirmed to be associated with adverse neonatal outcomes in preeclamptic twin pregnancies, then more attention should be given to the early detection and intervention of PE to improve the outcomes, and the evaluation of the indication of delivery should be made carefully.

### 4.2 Previous different results

As suggested above, we found that preeclamptic women with twin pregnancies had worse perinatal outcomes, as we predicted. Surprisingly, Sibai et al^[9]^ reported that women who remained normotensive throughout their pregnancy had a significantly lower gestational age at delivery, a lower birth weight, a higher rate of preterm delivery at both <37 and <35 weeks gestation and a higher rate of perinatal death. In fact, these results were partly from a multicenter randomized trial comparing low-dose aspirin with placebo for the prevention of PE. These women were randomly assigned aspirin or placebo at an early age, which probably had a potential influence on the pathological process and the perinatal outcomes. Moreover, some detailed information, such as the proportions of severe PE or early onset PE could not be obtained from the study, while 82.52% and 33.57% of the women were with severe PE and early onset PE in our study. The constitution of a severe form in the study population is also an influencing factor on the results. However, we thought that what the authors speculated was still reasonable, such that PE may represent a compensatory adaptive mechanism, such as promoting or restoring normal blood perfusion.^[^30–32^]^ Especially in preeclamptic twin pregnancies, more complex components are involved in its development and outcomes. Placental hypoperfusion is not the only explanation. The discrepant results between Sibai's and our study require further investigation.

### 4.3 Birth weight of twin pregnancy with PE

After adjusting for confounding factors, we still observed that the birth weight in the PE group was significantly lower, and the comparison of the discordance of the intertwin weight remained insignificant. Unique to multiple pregnancies is the phenomenon of discordant growth. Sparks et al^[33]^ demonstrated that women with hypertension (gestational hypertension and PE) increased the odds ratio of weight discordance ≥20% in the dichorionic twin. However, results from a relevant study were different, indicating that weight discordance was similar between the gestational hypertension and normotension groups in twin pregnancies. In addition, gestational hypertension may play a beneficial role in physiological processes and provided a better placental perfusion in twin pregnancies.^[34]^ According to these results, we thought the physiological mechanism of gestational hypertension might be unclear in twin pregnancies, so we excluded this subgroup in the present study. Finally, we found that, compared with normotensive women, PE was not associated with a higher discordance in the intertwin weight; although, maternal age, chorionicity, and gestational age at delivery were adjusted for in the analysis. Taking into consideration the worse neonatal outcomes in the preeclamptic mothers, we speculated that adverse neonatal outcomes in preeclamptic twin pregnancies were possibly the result of the lower birth weight rather than the weight discordance, which definitely requires further confirmation. Of note, we do not deny that the discordance is associated with a higher perinatal morbidity and mortality in other situations.^[35]^

The limitation of the present study could be the lack of information on some of the maternal factors, such as the maternal nutritional state, BMI, smoking behavior, and use of drugs. In addition, twin pregnancies in our tertiary care center may have been more likely to suffer from an adverse outcome compared to the average population. Meanwhile, the inability to make comparisons according to chorionicity was limited by a small sample in the monochorionicity group, so it is a challenge for us to obtain a larger population of twin pregnancies specific to chorionicity in the future.

In conclusion, twin pregnancies with PE are associated with worse perinatal outcomes. The adverse outcomes of preeclamptic twin pregnancies may be associated with lower birth weights rather than the discordance of the intertwin weight, which showed no differences between preeclamptic and normotensive women. The results from the present study may provide helpful references for better clinical assessments, evaluations of prognosis, and a deeper understanding in preeclamptic twin pregnancies.

## References

[1] Duley L The global impact of pre-eclampsia and eclampsia. *Semin Perinatol* 2009; 33:130–137. 19464502 10.1053/j.semperi.2009.02.010

[2] Bilano VL Ota E Ganchimeg T Risk factors of pre-eclampsia/eclampsia and its adverse outcomes in low- and middle-income countries: a WHO secondary analysis. *PLoS ONE* 2014; 9:e91198. 24657964 10.1371/journal.pone.0091198 PMC3962376

[3] Ye C Ruan Y Zou L The 2011 survey on hypertensive disorders of pregnancy (HDP) in China: prevalence, risk factors, complications, pregnancy and perinatal outcomes. *PLoS ONE* 2014; 9:e100180. 24937406 10.1371/journal.pone.0100180 PMC4061123

[4] Fox NS Roman AS Saltzman DH Risk factors for preeclampsia in twin pregnancies. *Am J Perinatol* 2014; 31:163–166. 23592316 10.1055/s-0033-1343775

[5] Sparks TN Cheng YW Phan N Does risk of preeclampsia differ by twin chorionicity? *J Matern Fetal Neonatal Med* 2013; 26:1273–1277. 23425367 10.3109/14767058.2013.777701

[6] Lučovnik M Tul N Verdenik I Risk factors for preeclampsia in twin pregnancies: a population-based matched case-control study. *J Perinat Med* 2012; 40:379–382. 22752768 10.1515/jpm-2011-0252

[7] Suzuki S Igarashi M Risk factors for preeclampsia in Japanese twin pregnancies: comparison with those in singleton pregnancies. *Arch Gynecol Obstet* 2009; 280:389–393. 19151988 10.1007/s00404-009-0932-4

[8] Krotz S Fajardo J Ghandi S Hypertensive disease in twin pregnancy: a review. *Twin Res* 2002; 5:8–14. 11893276

[9] Sibai BM Hauth J Caritis S Hypertensive disorders in twin versus singleton gestations. National Institute of Child Health and Human Development Network of Maternal-Fetal Medicine Units. *Am J Obstet Gynecol* 2000; 182:938–942. 10764477 10.1016/s0002-9378(00)70350-4

[10] Taguchi T Ishii K Hayashi S Clinical features and prenatal risk factors for hypertensive disorders in twin pregnancies. *J Obstet Gynaecol Res* 2014; 40:1584–1591. 24888920 10.1111/jog.12408

[11] Foo JY Mangos GJ Brown MA Characteristics of hypertensive disorders in twin versus singleton pregnancies. *Pregnancy Hypertens* 2013; 3:3–9. 26105734 10.1016/j.preghy.2012.05.005

[12] Henry DE McElrath TF Smith NA Preterm severe preeclampsia in singleton and twin pregnancies. *J Perinatol* 2013; 33:94–97. 22678139 10.1038/jp.2012.74

[13] Steegers EA von Dadelszen P Duvekot JJ Preeclampsia. *Lancet* 2010; 376:631–644. 20598363 10.1016/S0140-6736(10)60279-6

[14] Phoa KY Chedraui P Pérez-López FR Perinatal outcome in singleton pregnancies complicated with preeclampsia and eclampsia in Ecuador. *J Obstet Gynaecol* 2016; 20:1–4. 10.3109/01443615.2015.1107532 26790539

[15] Cunningham FG Leveno KJ Bloom SL Hypertensive Disorders, Preterm Labor. In: Cunningham FG Leveno KJ Bloom SL Spong CY Dashe JS Hoffman BL Casey BM Sheffield JS, eds. Williams Obstetrics, Sec. 11, 24th ed. New York, McGraw-Hill, 2014; 728–730, 829.

[16] Shetty A Smith AP The sonographic diagnosis of chorionicity. *Prenat Diagn* 2005; 25:735–739. 16170841 10.1002/pd.1266

[17] Senat MV Deprest J Boulvain M Endoscopic laser surgery versus serial amnioreduction for severe twin-to-twin transfusion syndrome. *N Engl J Med* 2004; 351:136–144. 15238624 10.1056/NEJMoa032597

[18] Ananth CV Vintzileos AM Shen-Schwarz S Standards of birth weight in twin gestations stratified by placental chorionicity. *Obstet Gynecol* 1998; 91:917–924. 9610996 10.1016/s0029-7844(98)00052-0

[19] Kingdom JC Nevo O Murphy KE Discordant growth in twins. *Prenat Diagn* 2005; 25:759–765. 16170859 10.1002/pd.1262

[20] Dikshit S Fresh look at the Doppler changes in pregnancies with placental-based complications. *J Postgrad Med* 2011; 57:138–140. 21654141 10.4103/0022-3859.81880

[21] Campbell S Placental vasculature as visualized by 3D power Doppler angiography and 3D colour Doppler imaging. *Ultrasound Obstet Gynecol* 2007; 30:917–920. 17960723 10.1002/uog.5195

[22] Sibley CP Turner MA Cetin I Placental phenotypes of intrauterine growth. *Pediatr Res* 2005; 58:827–832. 16183820 10.1203/01.PDR.0000181381.82856.23

[23] Fox NS Saltzman DH Oppal S The relationship between preeclampsia and intrauterine growth restriction in twin pregnancies. *Am J Obstet Gynecol* 2014; 211:422.e1–422.e5. 24881822 10.1016/j.ajog.2014.05.035

[24] Fox NS Rebarber A Klauser CK Intrauterine growth restriction in twin pregnancies: incidence and associated risk factors. *Am J Perinatol* 2011; 28:267–272. 21128199 10.1055/s-0030-1270116

[25] Wu D Huang L He Z Preeclampsia in twin pregnancies: association with selective intrauterine growth restriction. *J Matern Fetal Neonatal Med* 2015; 15:1–5. 10.3109/14767058.2015.1070140 26169709

[26] Derakhshi B Esmailnasab N Ghaderi E Risk factor of preterm labor in the west of Iran: a case-control study. *Iran J Public Health* 2014; 43:499–506. 26005661 PMC4433732

[27] Lu L Qu Y Tang J Risk factors associated with late preterm births in the underdeveloped region of China: A cohort study and systematic review. *Taiwan J Obstet Gynecol* 2015; 54:647–653. 26700979 10.1016/j.tjog.2014.05.011

[28] Kosińska KK Szymusik I Kaczyński B Iatrogenic and spontaneous late preterm twin—which are at higher risk of neonatal complications? *Ginekol Pol* 2013; 84:430–435. 24032260 10.17772/gp/1600

[29] Bastek JA Srinivas SK Sammel MD Do neonatal outcomes differ depending on the cause of preterm birth? A comparison between spontaneous birth and iatrogenic delivery for preeclampsia. *Am J Perinatol* 2010; 27:163–169. 19644790 10.1055/s-0029-1234036

[30] Seol HJ Cho GJ Oh MJ 2-Methoxyoestradiol levels and placental catechol-O-methyltransferase expression in patients with late-onset preeclampsia. *Arch Gynecol Obstet* 2013; 287:881–886. 23233290 10.1007/s00404-012-2663-1

[31] Sundrani DP Reddy US Joshi AA Differential placental methylation and expression of VEGF, FLT-1 and KDR genes in human term and preterm preeclampsia. *Clin Epigenet* 2013; 5:6. 10.1186/1868-7083-5-6 PMC3640948 23621880

[32] D'souza V Kilari A Pisal H Maternal nerve growth factor levels during pregnancy in women with preeclampsia: a longitudinal study. *Int J Dev Neurosci* 2015; 47:340–346. 26342999 10.1016/j.ijdevneu.2015.08.003

[33] Sparks TN Nakagawa S Gonzalez JM Hypertension in dichorionic twin gestations: how is birthweight affected? *J Matern Fetal Neonatal Med* 2016; 28:1–6. 10.3109/14767058.2016.1174209 27046743

[34] Ferrazzani S Moresi S De Feo E Is gestational hypertension beneficial in twin pregnancies? *Pregnancy Hypertens* 2015; 5:171–176. 25943640 10.1016/j.preghy.2015.01.003

[35] Breathnach FM Malone FD Fetal growth disorders in twin gestations. *Semin Perinatol* 2012; 36:175–181. 22713498 10.1053/j.semperi.2012.02.002

